# Evaluation of the University of California Diabetes Prevention Program (UC DPP) Initiative

**DOI:** 10.1186/s12889-021-11731-7

**Published:** 2021-09-30

**Authors:** Maryam Gholami, Nicholas J. Jackson, Un Young Rebecca Chung, O. Kenrik Duru, Kelly Shedd, Samantha Soetenga, Tamara Loeb, David Elashoff, Alison B. Hamilton, Carol M. Mangione, Wendelin Slusser, Tannaz Moin

**Affiliations:** 1 University of California, San Diego, San Diego, CA USA; 2 David Geffen School of Medicine at the University of California, Los Angeles, 1100 Glendon Ave, Suite 850, Los Angeles, CA 90024 USA; 3 Campus Recreation, University of California, Los Angeles, Los Angeles, CA USA; 4 VA Greater Los Angeles Healthcare System, Los Angeles, CA USA

**Keywords:** Diabetes prevention program, Implementation, RE-AIM

## Abstract

**Background:**

Type 2 diabetes can negatively impact long term health outcomes, healthcare costs and quality of life. However, intensive lifestyle interventions, including the Diabetes Prevention Program (DPP), can significantly lower risk of incident type 2 diabetes among overweight adults with prediabetes. Unfortunately, the majority of adults in the US who are at risk of developing diabetes do not engage in DPP-based lifestyle change programs. Increased adoption of evidence-based obesity and diabetes prevention interventions, such as the DPP, may help large employers reduce health risks and improve health outcomes among employees. In 2018, the University of California Office of thePresident (UCOP) implemented the UC DPP Initiative, a novel, multi-component program to address diabetes and obesity prevention across the UC system.

**Methods:**

The goal of our study is to conduct a multifaceted evaluation of the UC DPP Initiative using the Reach, Effectiveness, Adoption, Implementation, and Maintenance (RE-AIM) framework. Our evaluation will integrate unique and diverse UC data sources, including electronic health record (EHR) data, administrative claims, campus-based DPP cohort data, qualitative interviews and site visits. Our primary outcome of interest is the mean percent weight change among three groups of overweight/obese UC beneficiaries at risk for diabetes at 12-month follow-up. Secondary outcomes include mean percent weight change at 24-month follow-up, barriers and facilitators associated with implementatio, as well as  the degree of program adoption and maintenance.

**Discussion:**

Our study will help inform diabetes and obesity prevention efforts across the UC system. Findings from this evaluation will also be highly applicable to universities and large employers, as well as community organizers, healthcare organizations and insurers implementing the DPP and/or other health promotion interventions.

## Background

Type 2 diabetes is a chronic and often progressive disease that can lead to devastating complications and long-term disability [1]. The economic cost of diabetes is rising steeply, increasing from $245 billion in 2012 to $327 billion in 2017 [2]. The burden of diabetes on affected individuals and on society as a whole underscores the importance of prevention. The Centers for Disease Control and Prevention (CDC) estimate that up to 88 million adults aged 18 years or older have prediabetes, [1] and many of these individuals will progress to incident type 2 diabetes over 3 years without intervention [3, 4]. However, intensive lifestyle interventions (ILIs), including the Diabetes Prevention Program (DPP), can significantly lower risk of incident type 2 diabetes among overweight/obese adults with prediabetes [4]. Increased work-site adoption of obesity and diabetes prevention interventions, such as DPP-based ILIs, can help promote healthy weight among employees, [5, 6] reduce health risks, and improve health outcomes [6]. In a 2017 review of work-site translations of DPP, weight loss ranged between 1.4 and 4.9 kg at 7 to 12 months (*n* = 6 studies), with worksite programs offering > 16 core sessions having the most favorable outcomes [7].

**Fig. 1:**
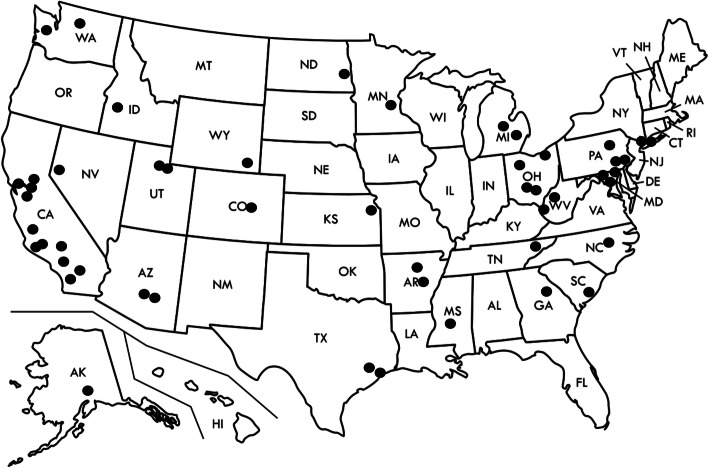
University-based Diabetes Prevention Program with full and preliminary recognition from the Centers for Chronic Disease Prevention and Control (CDC) https://nccd.cdc.gov/DDT_DPRP/Registry.aspx

Ongoing efforts to disseminate DPP nationally have increased the rate of program adoption by US employers, but reach and engagement among at-risk individuals remains relatively low [8]. University systems represent a promising, largely untapped option for DPP dissemination to overweight/obese adults with prediabetes. In the US, there are over 4300 higher education institutions (e.g., universities and colleges that grant degrees), [9] and in many communities, universities are the largest employer. The University of California (UC) system, for example, is one of the largest employers in California with over 229,000 employees [10]. University employee turnover may be lower than at for-profit organizations and many employees may also be enrolled in university-managed insurance programs, providing increased impetus to prevent diabetes and obesity. Large university systems also have many resources to readily implement DPP-based ILIs, making them an ideal setting to engage personnel in evidence-based obesity and diabetes prevention interventions. However, very few studies have examined the effectiveness of university-based DPP models. Among 1863 CDC registered DPP organizations as of March 1, 2021, 50 appear to be university or college-affiliated programs (Fig. 1). To our knowledge, only six published studies have focused on university based DPP adaptations. However, these studies included small sample sizes, pre-post analyses that lacked comparator groups, and short-term follow-up windows (Table 1) [11–16].

**Table 1:** Published DPP translation studies

Study, Year	Site	Study design	Outcomes of interest	N	Follow up	Mean weight loss	Implementation implications
﻿A Worksite Diabetes Prevention Program Two-Year Impact on Employee Health, 2006 [11]	﻿BD Medical, Sandy, Utah	﻿Within- group study design	﻿Weight, body mass index, waist circumference, oral glucose tolerance testing, fasting insulin, blood lipids, and aerobic fitness	22 (8 M, 14 F)	2 years	0.76 lbs.	N/A
*A Randomized Controlled Trial Translating the Diabetes Prevention Program to a University Worksite, Ohio, 2012–2014, 2016 [12]	﻿Ohio State University	Pretest–posttest control group design	﻿Primary outcome was percentage weight change; secondary outcomes were height, waist circumference, Biometrics, dietary intake, and PA levels	35	3 months	5.1 lbs.	﻿N/A
*Early Weight-Loss Success Identifies Non-responders after a Lifestyle Intervention in a Worksite Diabetes Prevention Trial, 2015 [13]	﻿Ohio State University	Pre and post intervention in randomized experimental group	﻿Percent weight loss	32 (5 M, 27 F)	4 months	﻿16.7 lbs. in those who lost > 5% weight & 5.6 lbs. in those who lost < 5% weight	N/A
*A translational worksite diabetes prevention trial improves psychosocial status, dietary intake, and step counts among employees with prediabetes: A randomized controlled trial, 2015 [14]	Ohio State University	﻿Randomized pretest/posttest control group design	﻿Psychosocial outcomes including self-efficacy for eating a low fat (LF) diet and PA, goal commitment and difficulty, employee ﻿presenteeism, social support, problem solving.	35	3 months	N/A	﻿N/A
Putting the National Diabetes Prevention Program to Work: Predictors of Achieving Weight-Loss Goals in an Employee Population, 2019 [15]	﻿Vanderbilt University Medical Center	﻿Exploratory analysis of data from DPP participants enrolled in the first 5 cohorts	﻿Primary outcome was characteristics and program metrics for DPP participants.	165	12 months		﻿Session attendance and physical activity independently predicted achievement of the 5% weight-loss goal in this worksite translation of the DPP.
Mixed methods study of engagement in behaviors to prevent type 2 diabetes among employees with pre-diabetes, 2016 [16]	﻿University of Michigan	Pre and post mixed methods study	﻿Facilitators of and barriers to engagement in recommended behaviors	82 (23 M, 59 F)	3 months	N/A	﻿Key facilitators of engagement included assistance and encouragement from social networks as well as use of external supports such as tracking devices. Barriers to engagement included competing demands and insufficient resources ﻿for healthy behaviors.

Beginning in 2018, the UC System  implemented the UC DPP Initiative, which is a multi-component program to target diabetes and obesity prevention among at risk affiliates. This initiative was informed by a pilot DPP implemented at the University of California Los Angeles (UCLA) in 2016. UCLA DPP was one of the first university-based programs in the US to achieve full CDC recognition. CDC recognition is granted to programs that meet all recommended milestones for DPP delivery, including 5% mean weight loss among participants. Based on the success of the UCLA campus-wide DPP, campus leaders and researchers partnered with the UC Office of the President (UCOP) and UC Health to launch a UC-wide DPP Initiative in 2018. By 2019, all 10 UC campuses had implemented UC DPP, which now operates as part of routine campus activities (more details below). To our knowledge, this is one of the largest university-based efforts to adress diabetes and obesity prevention with a focus on system-wide DPP-based ILI delivery.

Our goal is to conduct a rigorous, mixed-methods evaluation of the UC DPP Initiative, focusing on the five UC campuses with large medical centers where robust EHR data for UC beneficiaries is also available; namely UC Los Angeles, Irvine, San Diego, San Francisco, and Davis. Our goals are to identify why the UC DPP Initiative succeeds (or not), and to document lessons learned across the UC system and between campuses to inform future efforts across UC and other large university systems.

### The University of California Diabetes Prevention Program (UC DPP) initiative

The UC DPP Initiative has four key components; 1) identification of diabetes prevention as a system-wide goal, 2) a prediabetes awareness campaign targeting at-risk beneficiaries, 3) coordinated DPP implementation and delivery on every UC campus 4) coverage of DPP for all campus affiliates, including faculty, staff and students, at no cost. Each campus identified local champions, engaged key stakeholders, and signed a Memorandum of Understanding (MOU) with UCOP to receive funding for program implementation. The MOU outlined a commitment to the goals of the initiative, delineated guidelines for DPP delivery, and confirmed available resources. Funding to campuses was based on fixed costs of delivering a DPP cohort (i.e., not based on number of participants or program outcomes). Although the UC system could have easily continued to outsource DPP delivery (as many universities and workplaces do through their insurers), this initiative aimed to prioritize diabetes prevention as a highly visible system-wide goal that leverages university-based infrastructure and resources to implement all four key components. For example, campuses aimed to implement > 4 DPP cohorts in their first 2 years and then receive additional UCOP funding to add cohorts based on local demand.

The UC DPP Coordinating Center was established by UCOP/UC Health to support system-wide DPP activities. The UC DPP Coordinating Center assists UC campuses with all aspects of program delivery, including DPP coach training by UCLA-based certified master trainers and data management for DPP cohorts. Centralizing some activities, such as coach training, provides efficiency of scale (e.g., each individual campus is not required to spend time and money to train master trainers). The Coordinating Center leads monthly group calls with all UC campuses and individual calls with each campus as needed. Each UC campus is also registered with the National DPP (each campus has their own unique CDC organization number in order to be eligible for CDC recognition).

The UC system, like many other academic institutions, provides and manages health insurance benefits for many of its employees. The UC DPP Coordinating Center conducts outreach to at-risk UC health insurance beneficiaries with documented prediabetes (i.e., prediabetes diagnosed as part of usual care). Trained UCOP staff use UC EHR and claims data algorithms to identify at-risk participants with documented prediabetes. Personalized initiation letters with UC DPP logos are mailed from the Coordinating Center to overweight/obese UC beneficiaries with documented prediabetes. These prediabetes awareness letters include information about the importance of diabetes prevention and the UC DPP Initiative, including contact information for the local campus DPP. The letters clarify that there is no out-of-pocket cost for the UC DPP. UC DPP cohorts are led by UC staff who complete DPP coach training and have experience delivering campus-based wellness programs. DPP eligibility criteria include 1) age > 18 years, 2) body mass index (BMI) > 25 kg/m^2^ (> 23 for Asian adults) and 3) history of prediabetes, gestational diabetes, or a “high risk” CDC Prediabetes Risk Test. UC funding is provided to campuses for each additional DPP cohort and is not contingent on meeting National DPP metrics (e.g., 5% weight loss).

## Methods

To ensure a rigorous and multifaceted program evaluation, we will use the reach, effectiveness, adoption, implementation, and maintenance (RE-AIM) evaluation framework. The RE-AIM framework is a well-established tool to evaluate the implementation process of evidence-based health promotion programs and interventions, to address internal and external validity, as well as feasibility and generalizability [17–19]. We propose a rigorous, comprehensive evaluation of the UC DPP Initiative integrating unique and diverse data sources including EHR data, administrative claims, and campus data (DPP data, site visits and qualitative interviews)**.** Table 2 provides an overview of the UC DPP-specific metrics guided by the RE-AIM framework. Our study includes three specific aims described in more detail below.

**Table 2:** RE-AIM dimensions, questions and UC DPP-specific metrics

REAIM dimension & defining questions	UC DPP-specific metrics
Reach *What proportion of eligible participants a) were excluded, b) took part and c) how representative were those who participated?*	• Estimate persons reached based on the number eligible within each campus and across UC (received targeted outreach and enrolled in DPP) • Estimate persons engaged in DPP (attended > 9 and > 16 sessions) • Report exclusions, participation rates, drop-outs and representativeness within each campus and across UC
Effectiveness *What impact did the intervention have on a) all participants who began the program; b) on process and primary outcomes; and c) on both positive and negative (unintended) consequences?*	• Examine effectiveness (% weight change and DPP participation) within and between subjects (more detail below) • Examine consistency of outcomes across sites and key subgroups (e.g., race and ethnicity)
Adoption What proportion of stakeholders a) were excluded, b) participated and c) how representative were they?	• Assess representativeness of those making UC DPP related decisions with on each campus (i.e., those leading DPP efforts, implementation and maintenance) • Report types of key campus stakeholders involved within each campus and variations across UC
Implementation *To what extent were the various intervention components delivered as intended, especially when conducted by different non-research staff?*	• Assess facilitators and barriers to initiative implementation; examine how these vary across campuses and over time • Assess unintended consequences of implementation (i.e., support or resources pulled away from other programs) • Assess if UC DPP milestones are followed at all campuses or only partially implemented at some locations • Assess fidelity for at least two of 16 core DPP sessions at each campus • Assess similarities and differences in strategies across campuses (e.g., recruitment and engagement efforts, reported costs of program delivery)
Maintenance *To what extent was intervention maintained and what adaptations were required to maintain it? How was the original protocol modified? What was the attrition rate; were drop-outs representative?*	• Examine UC DPP outcomes within and across campuses, including attrition. • Report the degree to which UC DPP milestones were met over time • Report the degree to which initiative is integrated with campus workflow • Report whether the local and UC leadership provide upkeep and necessary support (e.g., staff)

*Adapted from RE-AIM [20, 21]

### Aim 1: effectiveness of UC DPP

To assess the effectiveness of the UC DPP Initiative, we will examine mean percent weight change from baseline at 12 month follow-up and 24 month follow-up within and between three groups of overweight/obese UC beneficiaries. Group 1 includes overweight/obese beneficiaries with documented prediabetes who receive UC DPP Initiative prediabetes awareness letters and enroll in DPP (i.e., receive “full treatment”). Group 2 includes overweight/obese beneficiaries with documented prediabetes who receive UC DPP Initiative prediabetes awareness letters, but do not enroll in DPP. Group 3 includes overweight/obese UC beneficiaries without documented prediabetes who are not included in the UC DPP Initiative (do not receive prediabetes awareness letters and do not enroll in DPP). Group 3 serves as a control group to account for secular trends and/or concurrent programs that may affect weight change outcomes among UC beneficiaries (such as weight management services any overweight/obese UC beneficiaries may receive as part of routine care). The primary outcome will be % weight change at 12-month follow-up within those who received a “full intervention dose” (i.e., Group 1) and secondary outcomes will compare mean % weight change between UC beneficiaries (Groups 1 vs. 2 and Group 1 + 2 vs. Group 3).

### Data collection & statistical analyses

To measure mean percent weight change, we will use EHR weights, which are collected similarly across UC health center visits, and DPP session weights for UC beneficiaries who enroll in DPP. Baseline covariate differences between those with and without attrition will be examined and we will then adjust for any covariates found to be significantly different (*p* < 0.05) between attrition groups in order to satisfy the Missing at Random assumptions of the mixed effects models. Because attrition could be a consequence of our primary outcome (i.e. weight) our missing data may be Missing Not at Random. In this instance we will utilize pattern mixture modeling to impute missing valuies. We will conduct within-subject analysis estimates of UC DPP Initiative effectiveness among those who received a “full intervention dose” (i.e., Group 1 prediabetes awareness letters and enrolled in DPP). For this primary analysis, we will use a mixed effects linear regression model to compare percent weight change within Group 1 subjects 12 and 24 months before DPP enrollment (pre period) and 12 and 24 months after enrollment (post period). Models for the trajectory of percent weight change will be specified with a random intercept for person with nested random slope for time from enrollment measured on a continuum. Average within-person between time-period (post vs. pre-enrollment) differences in the trajectory for % weight change will be modeled using a fixed effect interaction term of time-by-time-period to test for between time-period differences in the rate of % weight change. These models will be fit using a random intercept for person and random slope for time-period (post vs pre). A fixed effect of time-period will be used to estimate the average between time-period difference in % weight change adjusting for the corresponding length of time over which the change was observed. For both models, exploratory post-hoc analyses will also consider additional subject level covariates (age, gender, race, ethnicity, number of co-morbidities, number of DPP sessions attended, education level and years employed at the institution), as well as DPP site. Interactions of the difference over time (rates or mean levels) by these subject characteristics or site will also be explored to evaluate whether there is a differential benefit of the program for certain groups of beneficiaries (i.e., heterogeneity in treatment effect) and across sites. In the presence of statistically significant (*p* < 0.05) interactions, the models will be stratified so as to allow for potential heterogeneity in the covariates as well.

We will also compare outcomes between UC beneficiaries in Groups 1 vs. 2, a between-subject comparison, to estimate DPP effectiveness while holding the effect of the prediabetes awareness campaign constant across groups (both Group 1 and 2 receive prediabetes outreach letters). The mixed effects models mentioned above will be modified to examine between group differences in within-person change by incorporating an interaction term with Group membership. Similarly, we will compare outcomes in Groups 1 + 2 vs. Group 3, a between-subject comparison akin to an intent-to-treat (ITT) analytic approach since outcomes are being assessed among all UC beneficiaries with documented prediabetes eligible to receive the UC DPP Initiative in comparison to similarly overweight/obese individuals not exposed to the program. We will use propensity score matching to identify comparable overweight/obese beneficiaries with (Groups 1 + 2) and without (Group 3) documented prediabetes. The propensity score model will include baseline age, gender, race/ethnicity, BMI, and medical co-morbidities. Matching will be accomplished through use of the nearest-neighbor algorithm. Between group balance in the propensity scores and covariates will additionally be examined. While Groups 1 and 2 have an easily defined baseline for differentiating between pre- and post- diabetes awareness (i.e., enrollment or awareness letter date), Group 3 will have their baseline selected as corresponding to 12–18 months (or 24–30 months) prior to their most recent EHR entry.

### Sample size and power calculations

For our primary outcome of % weight change at 12-month follow-up within subjects, we will use 5% weight change to estimate power based on the 2002 DPP randomized controlled trial [4] as well as National DPP recognition standards [22]. With a proposed sample size of *N* = 50 using a two-sided alpha level of 0.05, we would demonstrate 80% power to detect a paired difference of at least 3.3% and have > 98% power to detect our target weight change of 5%. For our outcome of weight change at 12-month follow-up between subjects, we conservatively assume that the average % weight loss for those in Group 2 (prediabetes notified) and Group 3 (usual care) will be 2 and 0% respectively. These values will yield about a 3% difference in weight loss between our comparison groups. Using an independent samples t-test as a simplification of the mixed effects analysis plan with a two-sided alpha of 0.05, we will have 90% power to detect our assumed between group difference of 3% with the proposed sample sizes of 50 (Group 1; 10 per site) and 500 (Group 2; 100 per site). Using the same assumptions, the proposed sample sizes of *N* = 550 Group A + B and *N* = 500 Group 3 would yield 99% power to detect the assumed 3% difference in the percentage weight change between the groups. We would additionally have 80% power to minimally detect a between group difference of 2.5 and 1.1% with these sample sizes. All sample sizes account for an anticipated 20% loss to follow-up [7]. We will not be powered to detect differences in rates of incident type 2 diabetes, but we will examine trends over time using validated EHR and claims data algorithms [23, 24]. Metformin is not specifically highlighted in the UC DPP Initiative, but since some beneficiaries with prediabetes may also seek out this option, [4, 25] we will also examine rates of metformin uptake using prescription data available in the EHR.

### Aim 2: reach of UC DPP

To assess reach, we will report the proportion of eligible UC beneficiaries who engage in DPP (attend > 9 and > 16 sessions) and their representativeness of UC beneficiaries overall. Using DPP enrollment and participation data from each campus, we will assess the proportion of eligible participants who enroll in DPP, as well as rates of participation and engagement according to CDC standards (e.g., average number of sessions attended, proportion who completed > 9 and > 16 sessions, proportion with documented physical activity minutes, etc.). We will assess the representativeness of those who enrolled and participated in DPP as compared to the population overall, as well as the subset who were contacted for enrollment but chose not to enroll. We will also report exclusions, participation rates, drop-outs and representativeness within campuses and across UC.

We will examine the effectiveness of the DPP program (% weight change and DPP participation) within and between subjects. We will evaluate the consistency of outcomes across sites and key subgroups (e.g., race and ethnicity). We will also construct a logistic regression model to evaluate factors that predict whether subjects that receive the prediabetes letter will enroll in DPP. This is equivalent to the comparison of patient level factors between Group 1 and Group 2. First, we will use bivariate logistic regression models to evaluate subject level characteristics (e.g., age, gender, race, ethnicity, BMI, change in weight between the pre time point and baseline, income level) that predict the outcome. Next, we will construct a multivariable logistic regression model using a Least Absolute Shrinkage and Selection Operator (LASSO), [26] to identify the combination of factors that predict whether eligible subjects enroll in DPP (Group 1 vs. Group 2).

### *Aim 3:* adoption, implementation, and maintenance of UC DPP

To examine adoption, implementation, and maintenance, we will conduct qualitative interviews (at 12 and 24 months) to assess the degree of implementation, identifying barriers and facilitators associated with implementation, and unanticipated consequences. For adoption, we will assess representativeness of those making UC DPP-related decisions on each campus (i.e., those *leading* DPP efforts, implementation, and maintenance) and report types of key campus stakeholders involved within each campus and variations across UC. For implementation, we will assess facilitators and barriers to initiative implementation; examine how these vary across campuses and over time. We will also assess unintended consequences of implementation (e.g., support and resources pulled away from other programs), and if UC DPP milestones are followed at all campuses or only partially implemented at some locations. We will evaluate fidelity for at least two of 16 core DPP sessions at each campus and will assess similarities and differences in strategies across campuses (e.g., recruitment and engagement efforts, reported costs of program delivery). Research staff will observe DPP sessions and use standardized checklists to assess fidelity of DPP delivery. For maintenance, we will examine program outcomes within and across campuses, including attrition. We will report the degree to which the UC DPP milestones were met over time and the degree to which initiative is integrated with campus workflow. We will also report whether the local and UC leadership provide upkeep and necessary support (e.g., staff).

Relevant RE-AIM constructs will be used as an initial organizing framework for data coding and analysis. We will use matrix analysis methods [27] for rapid turn-around of the results, including aggregated site profiles, to share with UC leadership. In-depth analysis of the qualitative data will be conducted using ATLAS.ti. Initially, a top-level codebook will be developed for the baseline interviews based on the semi-structured interview guide, [28] Using a constant comparison analytic approach, this codebook will be elaborated upon based on emergent themes, and will be adjusted as each round of interviews is reviewed. Interviews will be compared within each campus, across campuses, across roles, and over time. Additional sources of qualitative data (i.e., field notes from site visits) will also be included in the data set and will be coded separately and in relation to the interview data.

This study was funded by NIH/NIDDK (1R01DK124503–01) and approved by the UCLA IRB (20–000357-AM-00001).

## Discussion

Increasing engagement in diabetes and obesity prevention is of paramount importance, but reach and engagement often fall short in real world settings [8]. Thus, studies evaluating real-world approaches to enhance DPP translation across diverse segments of the population are critically needed. Comprised of 10 unique campuses and serving as the third largest employer in California, the UC System is an ideal setting to conduct evaluations of system-wide initiatives to address diabetes and obesity prevention.

The UC DPP Initiative is a multi-component program implemented across UC as of 2018. By using the established RE-AIM framework and incorporating diverse data sources, we will provide a detailed understanding of the reach, effectiveness, adoption, implementation and maintenance of the UC DPP Initiative. While university-based diabetes and obesity prevention interventions are being implemented in UC and other settings, our study is one of the first rigorous evaluations to be conducted to date.

Studies have shown worksite lifestyle change programs can provide convenience and accessibility, which may help enhance individual-level engagement [6]. Co-worker social support may increase the likelihood of achieving desired outcomes, such as increases in physical activity, and cost-savings may be enhanced when focusing on employees at risk for chronic diseases such as obesity, cardiovascular disease, and diabetes [15]. Multi-component work-site interventions, that consider individual, organization and community factors, are more successful than single component programs [29]. Universities are worksites, where all of these factors are important considerations.

Universities are also the type of setting where affiliates (i.e., faculty and staff) may spend extended hours beyond the typical work-day (e.g., recreational or academic activities). Additional resources, such as campus recreation and wellness services, can be leveraged to more effectively deliver interventions at lower costs. For example, many universities already employ wellness and/or health promotion staff who can be trained to also deliver an on-site DPP. Thus, examining whether the UC DPP Initiative works and potential lessons learned across the UC system has many important implications. Findings from this study will help inform future diabetes and obesity prevention efforts across UC, as well as the implementation of DPP-based interventions at other universities and large, stable employers.

## Data Availability

The datasets generated and/or analyzed during the current study are not publicly available in accordance with the existing UC data use agreement. Data requests submitted to the corresponding author will need to be reasonable and undergo UC approval.

## Abbreviations

UCOP: University of California Office of President
UC: University of California
DPP: Diabetes Prevention Program
UC DPP: University of California Diabetes Prevention Program
CDC: Centers for Disease Control and Prevention
UCLA: University of California Los Angeles
EHR: Electronic Health Record
MOU: Memorandum of Understanding
ITT: Intent-to-treat
BMI: Body Mass Index
RE-AIM: Reach, effectiveness, adoption, implementation, and maintenance
